# Interpretable machine learning for prediction of leptomeningeal metastasis in lung adenocarcinoma: a multi-centre retrospective study via “Prompt” model

**DOI:** 10.3389/fonc.2026.1894987

**Published:** 2026-09-03

**Authors:** Xinyang Li, Jingxiang Su, Yitao Fan, Yingda Xie, Hongyan Liu, Tingting Wei, Ruoyu Yao, Renke Yu, Lu Zheng, Bo Sun, Xiangjun Qiu, Jiachun Sun

**Affiliations:** 1 Henan Key Laboratory of Cancer Epigenetics, Cancer Institute, The First Affiliated Hospital, College of Clinical Medicine, Henan University of Science and Technology, Luoyang, Henan, China; 2 Department of Radiation Oncology, Tianjin Medical University Cancer Institute & Hospital, Tianjin, China; 3 Department of General Practice, Sir Run Run Shaw Hospital, Zhejiang University School of Medicine, Hangzhou, Zhejiang, China; 4 Department of Medical Oncology, The Affiliated Cancer Hospital of Zhengzhou University and Henan Cancer Hospital, Zhengzhou, Henan, China; 5 College of Basic Medicine and Forensic Medicine, Henan University of Science and Technology, Luoyang, Henan, China

**Keywords:** leptomeningeal metastasis, lung cancer, predictive model, SHAP, Streamlit, Tabular Prior-data Fitted Network (TabPFN)

## Abstract

**Background:**

Leptomeningeal metastasis (LM) is a serious complication of lung adenocarcinoma (LUAD). Owing to the difficulty in early diagnosis and the limited clinical benefit of available treatments, affected patients experience extremely poor outcomes. Thus, the machine-learning-enhanced predictive framework Prompt was developed to optimise early clinical identification and prognostic assessment. This model was designed to determine major risk factors for LM in patients with LUAD, estimate the possibility of LM onset and predict survival, thereby supporting clinical decision-making via an early-warning system and accurate survival prediction.

**Methods:**

Clinical data of patients diagnosed with LUAD complicated by LM (LUAD-LM) between 1 January 2012 and 1 January 2025 were obtained from electronic medical databases across multiple medical centres. Embedded feature selection was conducted using tree-based feature importance ranking. Model performance was evaluated using the area under the receiver operating characteristic curve (AUC) to determine the most suitable machine-learning model. Shapley additive explanation (SHAP) values were applied to interpret the high-risk diagnostic model, and survival outcomes were examined using Cox proportional hazards regression.

**Results:**

Among 6,784 screened LUAD cases, 181 individuals met the inclusion criteria for patients with confirmed LUAD-LM. A high-risk diagnostic model for LUAD-LM was developed using the Tabular Prior-data Fitted Network (TabPFN), an approach recently published in *Nature*. In the test cohort, the model achieved an AUC of 0.842, with an accuracy of 0.880, a precision of 0.900, a recall of 0.947 and an F1 score of 0.923, reflecting consistently strong discriminatory ability. SHAP value analysis revealed that total CO_2_ on arterial blood gas, TTLS, indirect bilirubin, EGFR exon 21 L858R mutation status, Karnofsky Performance Status (KPS) score and serum Ca^2+^ levels were key contributors to LUAD-LM. For prognostic assessment, KPS score, serum Na^+^ levels and serum Cl^-^ concentrations were crucial determinants. A web-based calculator built on the Streamlit framework facilitated clinical application.

**Conclusion:**

Prompt framework delivers exploratory LM risk and survival estimations for Chinese patients with LUAD, serving as a preliminary risk-stratification reference rather than a universally applicable clinical diagnostic tool pending prospective external validation across diverse populations and healthcare systems.

## 1 Introduction

Leptomeningeal metastasis (LM) occurs when malignant tumour cells infiltrate the pia mater and subarachnoid space, disseminate throughout the cerebrospinal fluid (CSF), develop multifocal lesions and cause neurological impairment (1). LM most frequently results from aggressive malignancies such as lung cancer (e.g. lung adenocarcinoma [LUAD] and small-cell lung cancer), malignant melanoma and breast cancer (2). Evidence indicates that approximately 3%–5% of patients with non-small cell lung cancer (NSCLC) eventually develop LM. Among those with LUAD, the reported incidence was 1.4%–3%. The risk is markedly higher in patients with NSCLC harbouring ALK rearrangements or EGFR mutations, with LM incidence reaching 10.3% and 9.4%, respectively (3–6).

LM is one of the most invasive complications of LUAD, posing substantial diagnostic and therapeutic challenges (7). Diagnostic confirmation is often difficult. Available treatment options remain limited, and responses are typically unsatisfactory, leading to poor outcomes. Reportedly, the median overall survival (OS) in NSCLC-associated LM (NSCLC-LM) is generally < one year (3.6–11 months) (8). Current diagnostic approaches rely on clinical manifestations, neuroimaging and the cytological identification of tumour cells in the CSF. Initial symptoms such as headache, nausea, vomiting and lower-limb weakness are common but nonspecific, contributing to frequent missed diagnoses. Therefore, LM should be considered in patients with cancer exhibiting multifocal neurological involvement (9). Contrast-enhanced magnetic resonance imaging (MRI) of the brain and spine remains the gold standard for LM assessment (10). However, up to 30% of patients with LM are reported to have unremarkable MRI scans (11). Notably, positive MRI findings in patients with NSCLC-LM are often associated with a greater burden of central nervous system (CNS) disease, whereas negative scans may indicate comparatively limited involvement (12). These limitations emphasise that imaging alone is insufficient for reliable LM screening. Although cytological examination of CSF remains the diagnostic ‘gold standard’, its initial sensitivity is relatively low (<50%). Sensitivity improves to approximately 75%–85% with repeated sampling (13). However, in clinical practice, lumbar puncture is technically demanding and carries procedural risks. Consequently, patients are often hesitant to undergo repeated CSF collections. Poor compliance further complicates timely diagnosis. Integrating clinical and serum biochemical markers into a multimodal predictive framework may be a promising method for the early identification and accurate diagnosis of LM, improving prognosis and treatment outcomes.

The application of machine learning to clinical risk prediction has expanded rapidly; however, as Pennisi et al. systematically demonstrated, methodological quality and external validation remain major concerns across the literature. Many published models exhibit substantial heterogeneity in algorithms, performance reporting, and validation strategies, limiting their readiness for bedside translation. Meanwhile, Pinto et al. emphasised through comparative meta-analysis that although AI-driven risk scores often achieve encouraging discriminative ability, their added value over conventional clinical scoring systems requires rigorous, prospective head-to-head validation before widespread adoption. These insights directly shaped our study approach: we deliberately selected the recently published TabPFN foundation model, incorporated calibration and decision curve analyses to assess clinical utility, and deployed an accessible web-based interface, while explicitly acknowledging the exploratory nature of our findings and the necessity for multi-population external validation (14).

The increasing incidence of NSCLC-LM underscores the need for developing robust, individualised prediction models. Although several studies have explored risk models for LM secondary to lung cancer, there remain real challenges. Conventional models perform poorly in early-warning scenarios, and tools specifically tailored to LUAD-LM prediction remain underdeveloped. To address these challenges, this study utilised real-world data from multiple medical centres in China and incorporated one of the latest machine-learning advances published in *Nature*: the Tabular Prior-data Fitted Network (TabPFN) (15). Built on a Transformer architecture, TabPFN employs in-context learning to achieve exceptional performance in small-sample tabular tasks (datasets with ≤10,000 samples and ≤500 features), outperforming traditional machine-learning algorithms. Featuring data generation, density estimation, reusable embedding learning and fine-tuning, the model enhances predictive accuracy through learning complex patterns in high-dimensional data. Based on these advantages, we developed Prompt, the first intelligent model specifically designed for the early high-risk prediction of LM in patients with LUAD.

**Figure 1:**
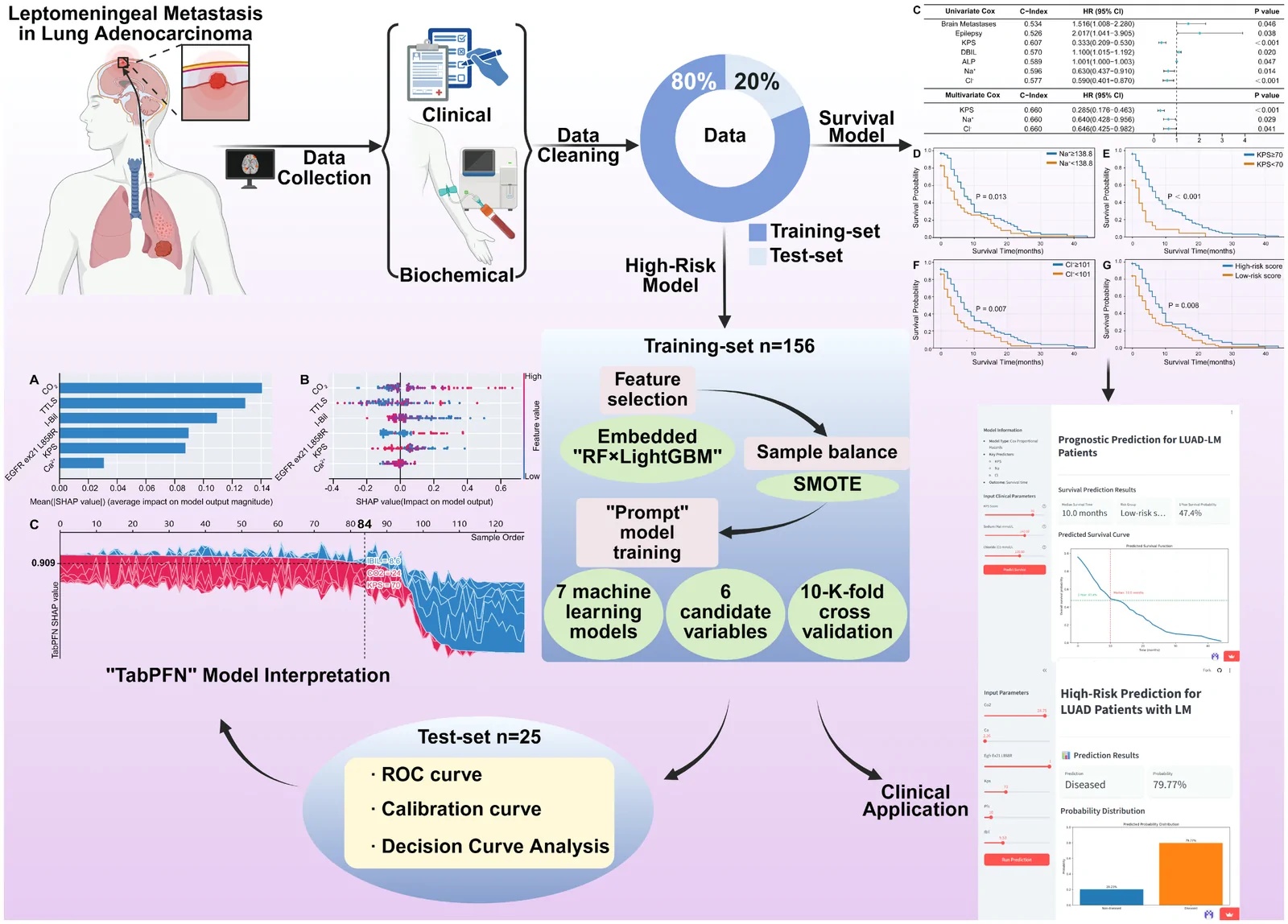
Schematic diagram.

This study addresses a methodological gap in the field and employs SHAP for model interpretability, overcoming the traditional ‘black box’ limitations associated with machine learning. The resulting analytical data provides clinicians with a clearer basis for decision-making. The model was deployed as a web-based tool using the Streamlit framework to facilitate practical clinical use. We anticipate that this study will enable earlier intervention and more accurate management of LUAD-LM. **Figure 1** shows the study’s overall technical workflow.

## 2 Methods

### 2.1 Study population and data sources

This retrospective study utilised de-identified electronic medical records from three major medical centres in China: Sir Run Run Shaw Hospital of Zhejiang University School of Medicine (Hangzhou); Henan Cancer Hospital, Affiliated Cancer Hospital of Zhengzhou University; and the First Affiliated Hospital of Henan University of Science and Technology (Luoyang).

Patients diagnosed with LUAD-LM between 1 January 2012 and 1 January 2025 were included. The inclusion criteria were age ≥18 years; histopathological confirmation of LUAD as the primary tumour; and LM diagnosis established by positive CSF cytology or characteristic MRI findings accompanied by clinical symptoms and/or CSF biochemical abnormalities. Conversely, the exclusion criteria included absence of pathological confirmation or presence of alternative histological subtypes (e.g. squamous cell carcinoma), insufficient evidence for LM (e.g. equivocal MRI findings without corroborating clinical or laboratory data) and incomplete clinical information or lacking follow-up data.

Clinical data were collected from electronic medical records, including all available parameters and serum biochemistry recorded during the patient’s initial hospitalisation until the end of data capture. The primary endpoint was survival outcomes during follow-up. The study protocol was approved by the ethics committees of the participating hospitals (Sir Run Run Shaw Hospital of Zhejiang University School of Medicine: approval no. 2025-2561-01; Henan Cancer Hospital, Affiliated Cancer Hospital of Zhengzhou University: 2025-775; and the First Affiliated Hospital of Henan University of Science and Technology [Luoyang]: approval no. 2024-1436). All procedures complied with relevant ethical requirements. Methodologically, the study adheres to the guidelines of the Transparent Reporting of a Multivariable Prediction Model for Individual Prognosis or Diagnosis (TRIPOD) statement (16).

### 2.2 Data preprocessing and feature selection

Feature selection relied on known etiological, pathological and therapeutic factors related to LUAD and LM, as well as the predefined inclusion and exclusion criteria. Variables with >20% missing data were excluded, yielding 50 features for model development and screening. All variables were extracted by experienced oncologists from the electronic medical records of the three participating centres.

The clinical variables included sex, age, smoking history, time to leptomeningeal suspicion (TTLS; defined as the interval from initial LUAD diagnosis to the first clinical suspicion of LM based on new neurological symptoms), and the use of antitumour agents, such as first- or third-generation EGFR-TKIs and bevacizumab. All predictor variables were measured at the last routine clinical assessment prior to the time of clinical suspicion of LM. Moreover, the following serum biochemical markers were analysed: albumin (ALB), globulin (GLB), albumin-to-globulin ratio, total bilirubin, direct bilirubin (DBIL), indirect bilirubin (IBIL), alanine aminotransferase, glucose, potassium, calcium (Ca^2+^) and carbon dioxide (CO_2_). LM risk stratification was based on positive or negative CSF cytology results, which served as the foundation for the high-risk diagnostic model. Subsequently, survival data were integrated to develop the prognostic model.

Mode imputation and multiple imputation were adopted to minimise the impact of missing data on classification performance. This hybrid approach preserves the underlying distributional characteristics of the dataset while ensuring completeness and internal consistency, providing a foundation for subsequent classification tasks. In addition, embedded feature selection was conducted using tree-based feature importance ranking, specifically combining the Random Forest (RF) and Light Gradient Boosting Machine (LightGBM) algorithms to identify highly relevant predictors. Furthermore, these algorithmically selected variables were investigated and consolidated based on clinical expertise and evidence to complete the feature-engineering workflow. Individuals without confirmed diagnostic records were excluded from the analytical cohort because the study focuses on assessing tumour cells in CSF and survival outcomes in patients with LUAD-LM. Then, the finalised features were merged into a unified dataset for further modelling.

The inclusion of features not consistently ranked in the top five by both algorithms was justified by established clinical and biological evidence, as well as their confirmed contribution to model predictions via SHAP importance analysis.

### 2.3 Model development, evaluation and interpretation

The dataset was randomly divided into a training cohort (80%) and an independent test cohort (20%) using a fixed random seed prior to any preprocessing or modelling steps. From the initial 129 real patient records, 104 patients were assigned to the training cohort and 25 to the test cohort. Critically, the test cohort was completely isolated from all subsequent analytical procedures and remained untouched throughout feature selection, hyperparameter tuning, and resampling. All feature engineering, 10-fold cross-validation, and SMOTE oversampling were performed exclusively within the training cohort to avoid data leakage. SMOTE was applied only to the minority class in the training set to address class imbalance during model training, and the final model was evaluated once on the entirely unmodified, real-patient test cohort.

The raw training cohort presented severe class imbalance, including 26 LM-negative and 78 LM-positive real patients. SMOTE was applied exclusively to the training subset to generate synthetic LM-negative minority samples via random interpolation based on the 5 nearest neighbours of each negative sample, with hyperparameters set as sampling_strategy=‘minority’, k_neighbors=5. After oversampling, the training set achieved a balanced 1:1 class distribution with 78 LM-negative and 78 LM-positive patients. The independent test cohort consisting of 6 LM-negative and 19 LM-positive authentic patients was completely excluded from all resampling procedures and retained unmodified raw clinical data. The generation volume of synthetic minority samples was moderately controlled to prevent artificial overestimation of model discriminative power. Repeated 10-fold cross-validation was performed on the balanced training set to confirm stable predictive performance.

The training set was utilised to develop machine-learning models, and the test set to evaluate predictive performance. The machine-learning algorithms used to develop the high-risk diagnostic model for LUAD-LM included logistic regression (LR), support vector machine (SVM), decision tree (DT), RF, extreme gradient boosting (XGBoost), LightGBM and TabPFN. Optimal hyperparameters for all seven models were determined through 10-fold cross-validation. Survival was predicted using the Cox proportional hazards regression model.

The performance of the LUAD-LM high-risk diagnostic model was assessed using multiple metrics, including accuracy, area under the receiver operating characteristic (ROC) curve (AUC), precision, recall, specificity, F1 score and Youden’s index. Defined as the harmonic mean of precision and recall, the F1 score provides a balanced metric particularly suitable for investigating datasets with class imbalance. Relying solely on either precision or recall yields misleading conclusions in such settings. A higher F1 score indicates more robust predictive power. ROC curves and AUC values were generated to compare the predictive performance of all models and identify the optimal algorithm. For the prognostic model, the predictive performance was evaluated using the concordance index (C-index) and Kaplan–Meier (KM) curve. Model interpretability was examined using SHAP, which ranks features according to their quantitative contribution to the prediction outcome. SHAP examines how each variable influences the model’s output, applying cooperative game theory to decrease the ‘black box’ nature of machine learning and facilitate clinical interpretation. Bar charts were utilised to visualise Global SHAP summaries, illustrating the average importance of each feature in the optimal prediction model. To support clinical translation, we developed a web-based calculator for the Prompt model and deployed it online via the Streamlit framework.

For the prognostic model, a Cox proportional hazards regression was refitted using clinically interpretable dichotomized predictors to satisfy the proportional hazards assumption. The final prognostic model was based on 129 real LUAD-LM patients (without SMOTE oversampling), with 117 death events and 12 censored cases. Missing data were handled using multiple imputation. Proportional hazards assumptions were verified using Schoenfeld residual-based tests. Internal validation was performed using bootstrap out-of-bag C-index estimation. Time-dependent AUC was assessed at 3, 6, and 12 months.

### 2.4 Statistical analysis

Patients with LUAD-LM were stratified into the CSF-positive and CSF-negative groups. Continuous data following normal distributions were presented as mean ± standard deviation ( $\overset{-}{x}$* ± s*), and non-normally distributed data as median and interquartile range. High-risk diagnostic models were established using all seven machine-learning algorithms. Survival analysis for the prognostic model was performed using the Cox proportional hazards regression. *P* < 0.05 was considered statistically significant. Analyses were conducted using R 4.2.1 or Python 3.8.10.

## 3 Results

### 3.1 Baseline characteristics of the study population

**Figure 2:**
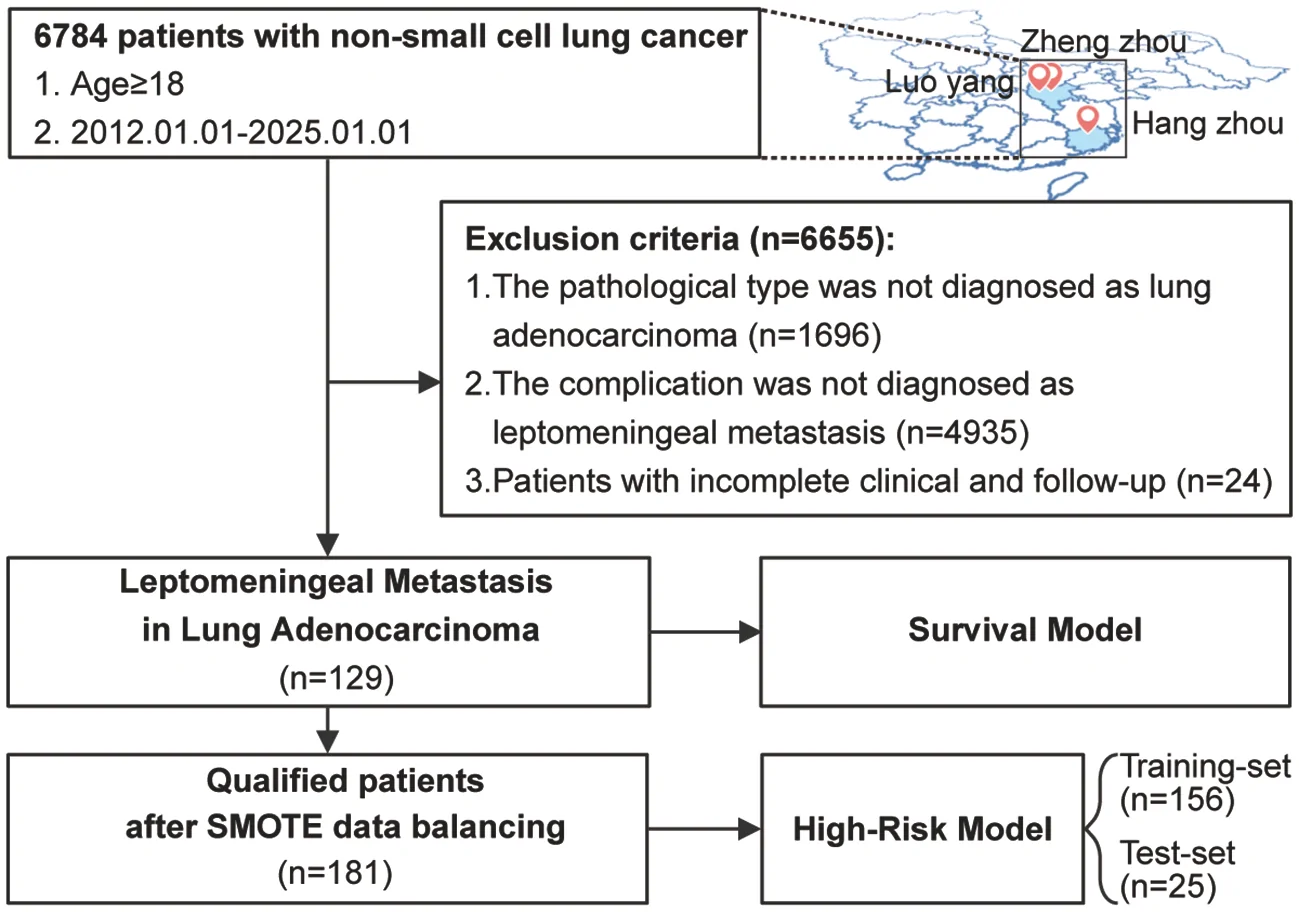
Schematic flowchart of subject enrolment.

Data of 6,784 patients with NSCLC were extracted from the electronic medical record databases of three medical centres. After applying strict inclusion and exclusion criteria, 129 real patients were included and randomly divided into the training cohort (n = 104) and independent test cohort (n = 25). SMOTE oversampling was subsequently applied only to the training cohort, resulting in 156 balanced training samples for model development (**Figure 2**). **Table 1** summarises the baseline characteristics of patients diagnosed with LUAD-LM after SMOTE-based data balancing, including clinical information, serum biochemical laboratory parameters, gene mutation profiles and the use of antitumour agents. The distribution of baseline characteristics was well-balanced and comparable between the training and test cohorts. Notably, 84 patients (46.4%) were classified as low-risk and 97 (53.6%) as high-risk. The training cohort included 78 low-risk and 78 high-risk patients. The median TTLS was 13.5 months in the training cohort and 25 months in the validation cohort.

**Table 1:** Baseline characteristics of LUAD-LM patients after SMOTE algorithm data balancing.

Characteristics	Overall	Training-set	Test-set	P-value	Method
	N=181	N=156	N=25		
CSF (%)				0.016	Chisq test
Positive	97 (53.6)	78 (43.1)	19 (10.5)		
Negative	84 (46.4)	78 (43.1)	6 (3.3)		
Gender (%)				0.902	Chisq test
Male	56 (30.9)	48 (26.5)	8 (4.4)		
Female	125 (69.1)	108 (59.7)	17 (9.4)		
Age (median[IQR])	59 (53, 67)	58 (52, 67)	64 (60, 67.5)	0.806	Wilcoxon
Smoking (%)				1.000	Yates’ correction
Yes	29 (16.0)	25 (13.8)	4 (2.2)		
No	152 (84.0)	131 (72.4)	21 (11.6)		
TTLS (median[IQR])	14 (5, 30)	13.5 (4.75, 28.25)	25 (10, 35)	0.092	Wilcoxon
Tumour, T (%)				0.767	Chisq test
T_3-4_	70 (38.7)	61 (33.7)	9 (5.0)		
T_1-2_	111 (61.3)	95 (52.5)	16 (8.8)		
Node, N (%)				0.003	Chisq test
N_0-1_	39 (21.5)	28 (15.5)	11 (6.1)		
N_2-3_	142 (78.5)	128 (70.7)	14 (7.7)		
Metastasis, M (%)				1.000	Yates’ correction
M_1_	156 (86.2)	134 (74.0)	22 (12.2)		
M_0_	25 (13.8)	22 (12.2)	3 (1.7)		
Prior Staging (%)				0.082	Yates’ correction
III, IV	173 (95.6)	151 (83.4)	22 (12.2)		
II	4 (2.2)	2 (1.1)	2 (1.1)		
I	4 (2.2)	3 (1.7)	1 (0.6)		
EGFR ex21 L858R (%)				0.279	Chisq test
Mutation	49 (27.1)	40 (22.1)	9 (5.0)		
Wild-type	132 (72.9)	116 (64.1)	16 (8.8)		
TKI Therapy (%)				0.097	Chisq test
Yes	135 (74.6)	113 (62.4)	22 (12.2)		
No	46 (25.4)	43 (23.8)	3 (1.7)		
Bevacizumab (%)				0.262	Chisq test
Yes	42 (23.2)	34 (18.8)	8 (4.4)		
No	139 (76.8)	122 (67.4)	17 (9.4)		
TKI Efficacy (%)				0.651	Yates’ correction
PD/SD	105 (58.0)	90 (49.7)	15 (8.3)		
Non	33 (18.2)	30 (16.6)	3 (1.7)		
PR	43 (23.8)	36 (19.9)	7 (3.9)		
Brain Metastases (%)				0.705	Chisq test
Yes	117 (64.6)	100 (55.2)	17 (9.4)		
No	64 (35.4)	56 (30.9)	8 (4.4)		
Dizziness (%)				0.272	Chisq test
Yes	53 (29.3)	48 (26.5)	5 (2.8)		
No	128 (70.7)	108 (59.7)	20 (11.0)		
Headache (%)				0.145	Chisq test
No	130 (71.8)	109 (60.2)	21 (11.6)		
Yes	51 (28.2)	47 (26.0)	4 (2.2)		
Consciousness (%)				0.697	Chisq test
Normal	52 (28.7)	44 (24.3)	8 (4.4)		
Abnormal	129 (71.3)	112 (61.9)	17 (9.4)		
Myelopathy (%)				0.232	Yates’ correction
Yes	20 (11.0)	15 (8.3)	5 (2.8)		
No	161 (89.0)	141 (77.9)	20 (11.0)		
KPS (median[IQR])	70 (70, 80)	70 (70, 80)	70 (70, 80)	0.806	Wilcoxon
TP (median[IQR])	69.73 (65.40, 73.06)	69.98 (65.42, 73.06)	68.80 (61.20, 75.40)	0.354	Wilcoxon
ALB (median[IQR])	41.90 (38.50, 43.80)	41.90 (38.68, 43.72)	41.70 (36.30, 44.30)	0.866	Wilcoxon
GLB (median[IQR])	27.25 (25.00, 29.40)	27.40 (25.34, 29.47)	25.80 (24.40, 29.30)	0.220	Wilcoxon
A/G (median[IQR])	1.48 (1.30, 1.70)	1.46 (1.30, 1.69)	1.50 (1.33, 1.70)	0.414	Wilcoxon
TBIL (median[IQR])	10.00 (7.41, 13.40)	9.95 (7.40, 13.40)	10.50 (7.70, 13.60)	0.642	Wilcoxon
DBIL (median[IQR])	3.50 (2.70, 4.48)	3.60 (2.80, 4.49)	3.00 (2.40, 4.30)	0.290	Wilcoxon
IBIL (median[IQR])	7.59 (5.40, 8.96)	7.70 (5.47,9.00)	7.30 (5.40, 8.50)	0.876	Wilcoxon
ALT (median[IQR])	18.00 (12.72, 25.00)	18.16 (13.00, 25.00)	18.00 (11.00, 25.00)	0.374	Wilcoxon
AST (median[IQR])	19.00 (16.24, 24.00)	19.00 (16.35, 24.00)	19.00 (16.00, 24.00)	0.837	Wilcoxon
ALP (median[IQR])	75.00 (31.00, 95.00)	75.00 (33.86, 95.15)	68.00 (23.00, 94.00)	0.685	Wilcoxon
UREA (median[IQR])	4.30 (3.60, 5.46)	4.25 (3.60, 5.39)	4.40 (3.60, 5.80)	0.535	Wilcoxon
CREA (median[IQR])	63.00 (54.00, 70.00)	63.00 (54.00, 69.12)	63.00 (50.00, 83.99)	0.805	Wilcoxon
URCA (median[IQR])	251.87 (195.00, 303.00)	251.87 (189.00, 289.15)	252.00 (212.00, 322.00)	0.439	Wilcoxon
GLU (median[IQR])	5.54 (4.95, 7.47)	5.57 (4.99, 7.74)	5.32 (4.58, 6.34)	0.191	Wilcoxon
K^+^ (median[IQR])	4.08 (3.94, 4.39)	4.08 (3.95, 4.41)	4.02 (3.80, 4.24)	0.163	Wilcoxon
Ca^2+^ (median[IQR])	2.29 (2.17, 2.53)	2.31 (2.18, 2.61)	2.22 (2.12, 2.30)	**0.017**	Wilcoxon
Na^+^ (median[IQR])	138.00 (137.17, 140.93)	138.00 (137.37, 140.49)	140.00 (136.60, 141.50)	0.153	Wilcoxon
Cl^-^ (median[IQR])	102.12 (100.00, 104.50)	102.12 (100.28, 104.15)	102.10 (99.10, 106.30)	0.998	Wilcoxon
CO_2_ (median[IQR])	24.00 (22.72, 25.34)	24.00 (22.47, 25.00)	25.00 (23.30, 26.00)	**0.029**	Wilcoxon

Bold values represent categorical variables.

### 3.2 Feature selection for model development

Spearman’s correlation analysis (**Supplementary Figure S1**) was applied to ensure that the features included in the subsequent high-risk diagnostic model were not highly collinear, avoiding multicollinearity that may impair model fitting.

In the training cohort, embedded feature selection was applied. RF and LightGBM algorithms were employed to rank the relevance of the initial 50 variables (**Supplementary Figures S2**, **S3**). The top five features by importance after processing were selected to form Feature Set ‘RF’ and Feature Set ‘LightGBM’, respectively. Based on clinical experience and previous literature, five variables (i.e. TTLS, KPS, EGFR exon 21 L858R mutation status, CO_2_ and Ca^2+^), which are potentially associated with an increased risk of developing LM in patients with LUAD, were included in the modelling process as Feature Set ‘Tendency’.

**Figure 3:**
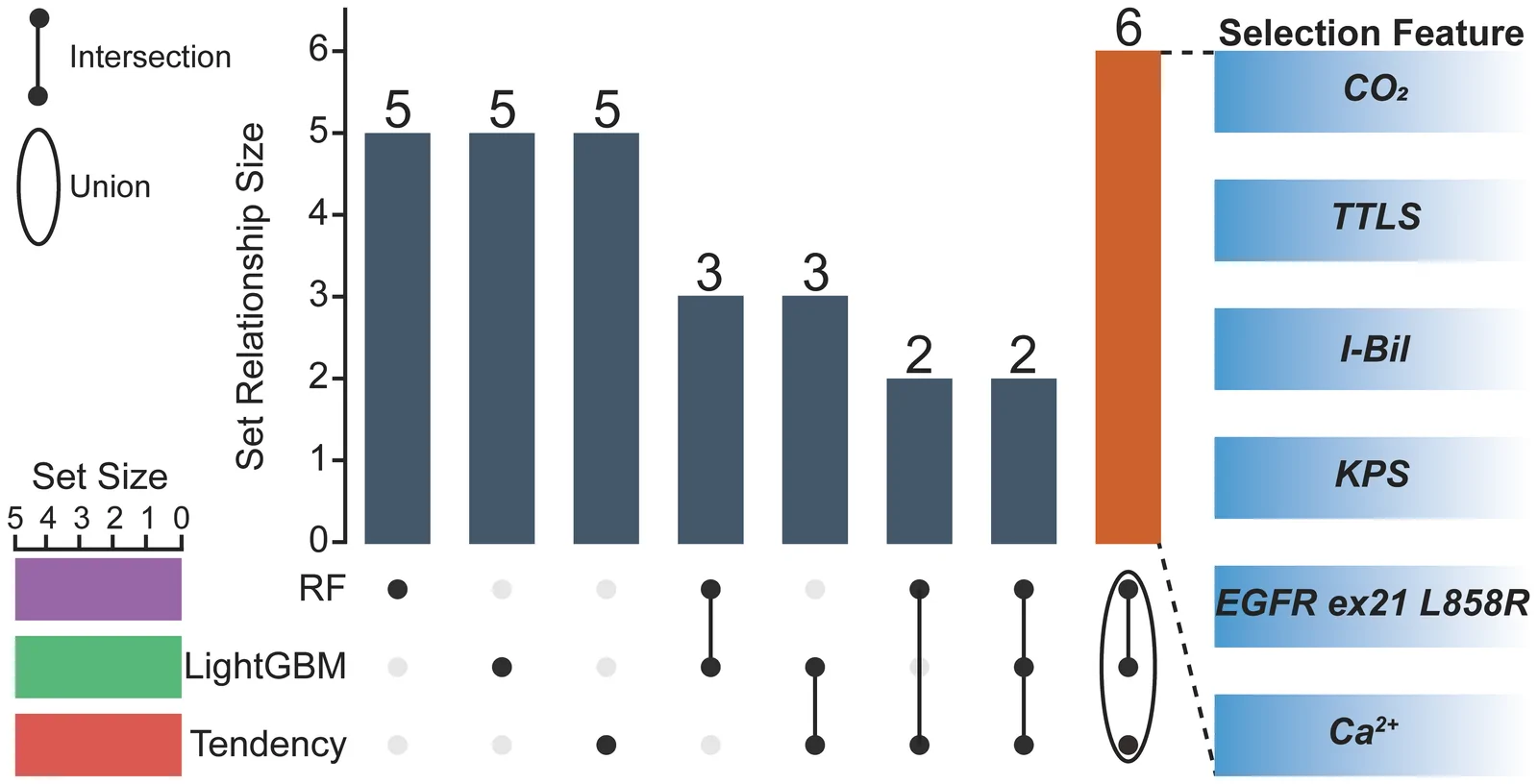
UpSet plot representation of feature engineering.

Feature Set ‘RF’ and Feature Set ‘LightGBM’ yielded three common variables. These were combined with Feature Set ‘Tendency’ to form a union set; thus, six final predictive features were incorporated into the model (**Figure 3**).

After processing, the original 50 variables were decreased to 6 predictive features for inclusion in the LM high-risk diagnostic model. These six variables involved clinical information and serum biochemical markers, including total CO_2_ on arterial blood gas (ABG) analysis, TTLS, IBIL, EGFR exon 21 L858R mutation status, KPS and serum Ca^2+^ (**Table 1**). Rigorous univariate and multivariate Cox regression analyses identified three highly prognostic variables, which were incorporated as predictive features in the prognostic model.

### 3.3 Development and validation of the predictive model

**Figure 4:**
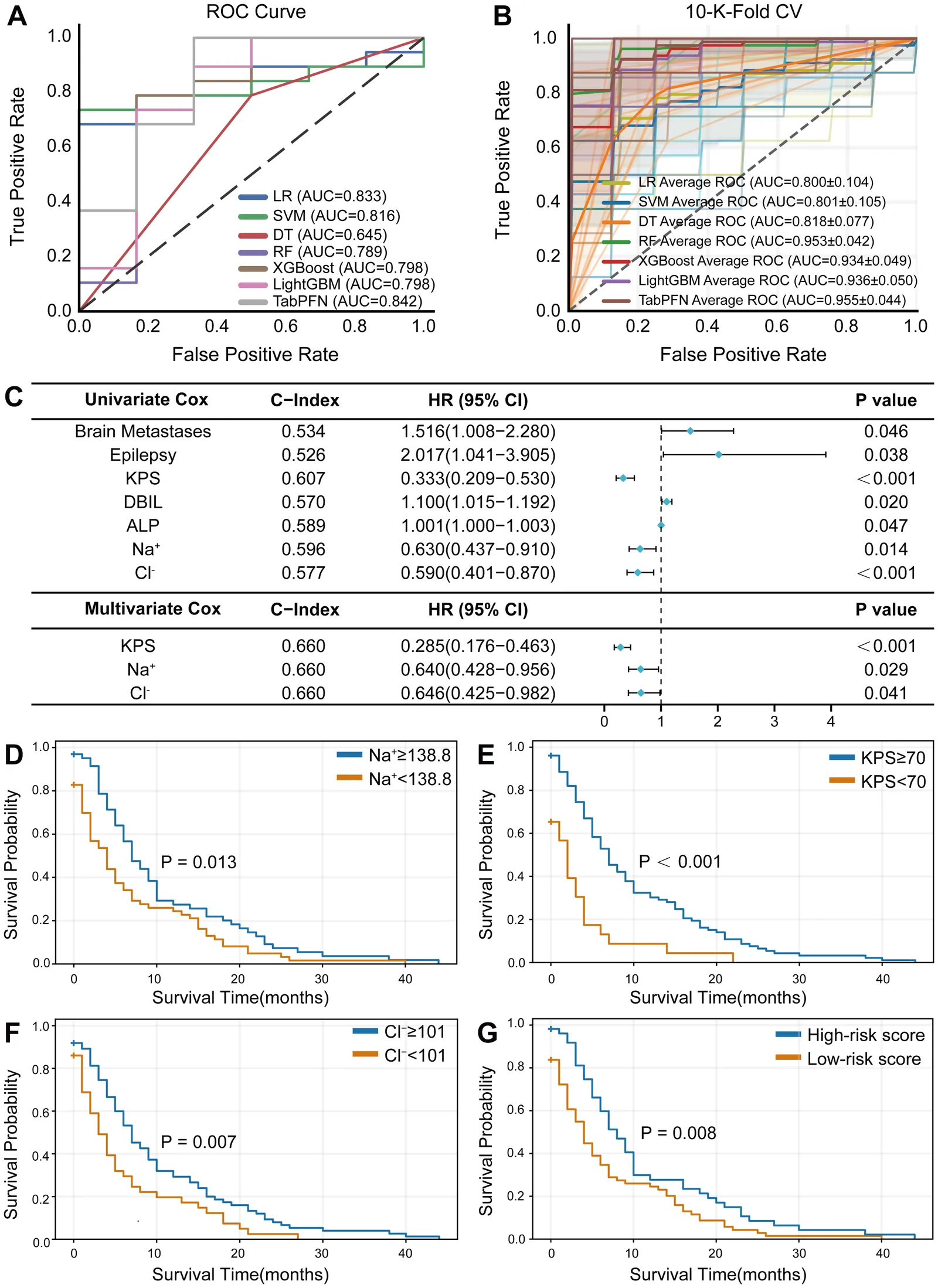
Development and validation of the predictive model: **(A)** ROC curves of the seven machine-learning models in the test set; **(B)** Average ROC curves from the 10 fold cross-validation internal validation of the seven machine-learning models; **(C)** Forest plot of univariate and multivariate Cox regression analysis for survival; **(D–G)** Kaplan-Meier survival curves for patient subgroups.

After calculating and tuning the relevant parameters, the optimal hyperparameter settings for the seven machine-learning algorithms were determined (**Table 2**). Following hyperparameter optimisation, all seven models were trained using the full training dataset. Their performance was comprehensively evaluated in the independent test cohort. The ability of each model to discriminate patients at high risk for LM was examined using ROC curve analysis. The AUC values for LR, SVM, DT, RF, XGBoost, LightGBM and TabPFN were 0.833, 0.816, 0.645, 0.789, 0.798, 0.798 and 0.842, respectively (**Figure 4A**). To obtain a more multidimensional picture of the model’s behaviour, precision, recall, specificity, F1 score and Youden’s index were calculated. Furthermore, the relative performance across models was visualised using radar plots (**Table 3**). Considering the AUC values together with the outcomes of the 10-fold cross-validation, the TabPFN model consistently achieved the highest AUC values across the primary evaluation and cross-validation analyses. Thus, it was identified as the most robust risk predictor of LUAD-LM (**Figure 4B**). In the test set, the TabPFN model showed an accuracy of 0.880, a precision of 0.900, a recall of 0.947 and an F1 score of 0.923, demonstrating excellent predictive power. The Hosmer–Lemeshow goodness-of-fit test yielded a *P*-value of 0.907 (>0.05), indicating that the model fits the observed data well. Underfitting or overfitting was not detected, suggesting that the final model provides a reliable representation of the underlying clinical patterns. Beyond discrimination performance, we further comprehensively evaluated the calibration consistency and clinical practicability of the TabPFN model (**Supplementary Figure 4**). In the independent test cohort, quantitative calibration metrics were calculated as follows: a Brier score of 0.111, a calibration intercept of 0.104, and a calibration slope of 0.848. These indicators collectively demonstrate reasonable concordance between model-predicted LM risk probabilities and actual observed event rates. We additionally performed decision curve analysis (**Supplementary Figure 5**) to quantify clinical net benefit across continuous threshold probabilities. Within the threshold range of approximately 0.2 to 0.8, the TabPFN model generated superior net benefit compared with both treat-all and treat-none reference strategies, which confirms that the model can provide clinically meaningful risk stratification rather than merely reliable discriminative ability.

**Table 2:** Optimal hyperparameters of the seven machine-learning prediction models.

Model	Optimal parameter
LR	penalty=I2, C = 1, class_weight=balanced
SVM	penalty=I2, C = 1, class_weight=balanced, loss=squared_hinge
DT	criterion=gini
RF	criterion=gini, n_estimators=100, oob_score=FALSE
XGBoost	learning_rate=0.1, max_depth=3, n_estimators=100
LightGBM	learning_rate=0.1, max_depth=-1, n_estimators=100
TabPFN	model: tabpfn-v2-classifier-finetuned-llderlii-oyd7ul21.ckpt, n_estimators=28

**Table 3:** Predictive performance of the seven machine-learning models in test set.

Model	AUC	Accuracy	Precision		Recall	Specificity
LR	0.833	0.760	0.882		0.789	0.667
SVM	0.816	0.720	0.833		0.789	0.500
DT	0.645	0.720	0.833		0.789	0.500
RF	0.789	0.840	0.857		0.947	0.500
XGBoost	0.798	0.800	0.850		0.895	0.500
LightGBM	0.798	0.840	0.857		0.947	0.500
**TabPFN**	**0.842**	**0.880**	**0.900**		**0.947**	**0.667**
Model	F1	Youden’s Index		Radar Chart		
LR	0.833	0.456		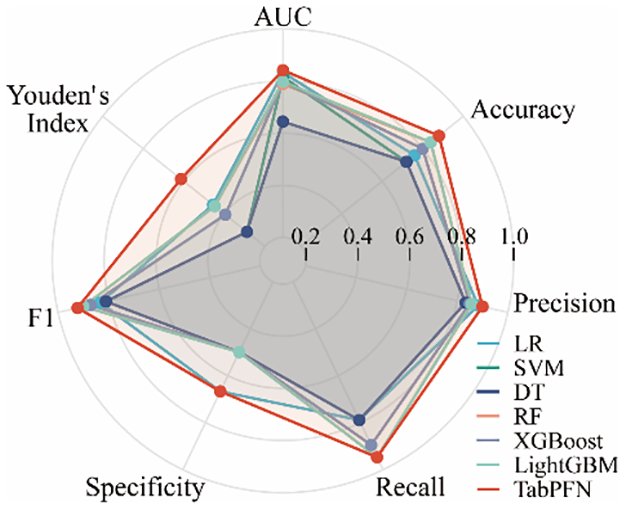		
SVM	0.811	0.289				
DT	0.811	0.289				
RF	0.900	0.447				
XGBoost	0.872	0.395				
LightGBM	0.900	0.447				
**TabPFN**	**0.923**	**0.614**				

Bold values represent performance evaluation metrics of the TabPFN model.

For prognostic assessment, the Cox model was refitted using dichotomized predictors to satisfy the proportional hazards assumption. The final model included KPS≥70, serum Na^+^≥138.8 mmol/L, and serum Cl^-^≥101 mmol/L. On multivariable analysis, KPS≥70 (HR = 0.285, 95% CI: 0.176–0.463, P<0.001), Na^+^≥138.8 mmol/L (HR = 0.640, 95% CI: 0.428–0.956, P = 0.029), and Cl^-^≥101 mmol/L (HR = 0.646, 95% CI: 0.425–0.982, P = 0.041) were independently associated with improved overall survival. The model C-index was 0.660, and Schoenfeld residual-based tests showed no evidence of proportional hazards violation (KPS: P = 0.0695; Na^+^: P = 0.0630; Cl^-^: P = 0.8874). Internal validation via bootstrap out-of-bag C-index yielded a mean of 0.652 (95% range: 0.566–0.729). Time-dependent AUC values at 3, 6, and 12 months were 0.736, 0.703, and 0.592, respectively (**Supplementary Figure 6**), indicating favourable short-term discriminative performance but more limited long-term prediction. Kaplan-Meier curves demonstrated significant survival stratification based on each individual predictor (all log-rank P<0.05) and, importantly, based on the full multivariable risk score (high-risk vs. low-risk, log-rank P = 0.008; median OS 4 vs. 8 months) (**Figures 4D–G**). These results collectively support the model’s utility for short-to-medium term prognostic stratification in LUAD-LM patients.

### 3.4 Model interpretation

**Figure 5:**
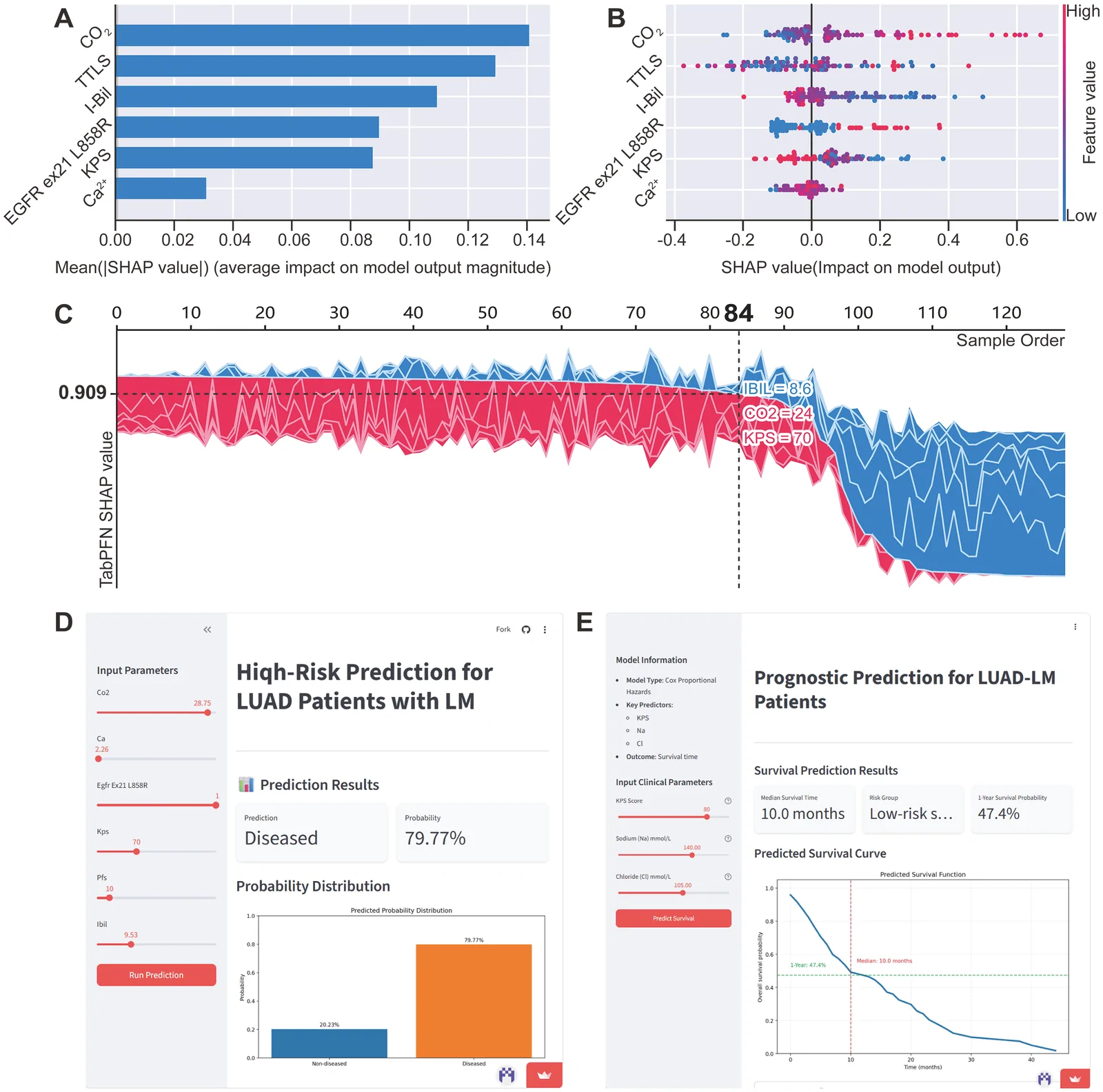
Interpretation of the predictive model: **(A)** SHAP bar plot. The horizontal axis represents the mean absolute SHAP value of each feature, indicating its overall importance to the model’s predictions; **(B)** SHAP summary plot. The distribution of SHAP values for each feature is displayed, where colour denotes the original feature value (red for high, blue for low) and the x-axis position represents the SHAP value’s magnitude and direction; **(C)** SHAP force plot. This illustrates the contribution (SHAP value) of each feature to the prediction for an individual sample. **(D, E)** A web-based calculator deployed using the Streamlit framework, corresponding to the high-risk model and the prognostic model for LUAD-LM patients, respectively.

SHAP value analysis was conducted to determine the influence of each predictor on the model’s output. SHAP values represent the extent to which individual features shift the final prediction, providing a transparent view of why a patient is assigned a given risk estimate. In our interpretation framework, six variables emerged as the most influential determinants of high-risk cases: total CO_2_ on ABG analysis, TTLS, IBIL, EGFR exon 21 L858R mutation status, KPS and serum Ca^2+^. These risk factors were ranked based on their mean absolute SHAP values (**Figure 5A**). The SHAP summary plot reveals the relationship between feature magnitude and SHAP value. A larger SHAP value indicates a stronger effect of that variable on the TabPFN model’s predictions. Data points in red mark higher feature values, while blue dots denote lower values. Overall, increased total CO_2_, longer TTLS, presence of the EGFR L858R mutation, higher serum Ca^2+^ levels, lower IBIL concentrations and decreased KPS scores showed the strongest associations with increased risk of LM in patients with LUAD (**Figure 5B**). To explore individualised interpretability, SHAP force plots for the TabPFN model were also generated. These individualised visualisations reveal how specific features shape risk estimates. In patient no. 84, the model incorporated inputs such as IBIL = 8.6 μmol/L, CO_2_ = 24 mmol/L and KPS score = 70, yielding a computed SHAP value of 0.909 (**Figure 5C**).

An online calculator was developed for the prediction models using the Streamlit framework in Python; the application was deployed on the Web. The interface for estimating the risk of LM in patients with LUAD can be accessed at https://leptomeningealmetastasis-fu4evnbchcxqsgp9rjbwdjv2.streamlit.app/, and the interface for prognostic assessment in patients with LUAD is available at https://frylmu9rezow9wjda34uihv2.streamlit.app/. These tools were established to support real-world clinical use. The TabPFN-based calculator, which incorporates six predictors, enables clinicians to estimate LM risk for patients with LUAD. When the actual values of these six variables are entered, the calculator provides an estimated probability of LM (i.e. 79.77% in the example) (**Figure 5D**). For prognostic assessment, if a patient with LUAD-LM has a KPS score of 80, serum Na^+^ of 140 mmol/L and serum Cl^-^ of 105 mmol/L, the model predicts a median survival of approximately 10 months and a 1-year survival probability of 47.4% (**Figure 5E**).

## 4 Discussion

LM is a fatal complication of LUAD. The lack of distinctive clinical manifestations or reliable early diagnostic methods leads to extremely poor survival outcomes in these patients (17). Accurate prediction models are crucial for identifying high-risk individuals early, optimising treatment strategies and supporting prognostic assessment. Although several prediction models have been developed to evaluate the possibility of LM in lung cancer, real-world studies on applying machine learning to characterise LM risk and survival in LUAD remain limited. To address this gap, a multicentre retrospective analysis was conducted to analyse the clinical characteristics and risk factors of LUAD-LM using machine-learning approaches. Among the seven machine-learning models evaluated, the TabPFN algorithm demonstrated the strongest performance (AUC = 0.842).

Despite notable progress in assessing the risk of LM in lung cancer, existing models still lack sufficient predictive accuracy. Positioning our model within the contemporary AI-prediction landscape, we recognise that although machine learning has been increasingly applied to cancer metastasis prediction, the specific niche of leptomeningeal metastasis in lung adenocarcinoma remains underexplored, particularly with regard to foundation models optimised for small-sample tabular data. Our adoption of TabPFN, as highlighted by Pennisi et al. (18), represents a methodological step beyond conventional tree-based or regression classifiers, addressing the need for novel architectures in rare-disease contexts. However, consistent with Pinto et al. (14), we caution that encouraging internal performance metrics should not be equated with clinical readiness. The true test of our model lies in prospective comparisons against established nomograms, CSF cytology-driven algorithms, or clinician judgement in real-world diagnostic pathways. We therefore reiterate that the Prompt model, in its current form, offers an exploratory risk-stratification reference rather than a universally applicable diagnostic tool, and its integration into clinical practice must await rigorous external validation across diverse healthcare systems and populations. Li et al. established an LM diagnostic model using traditional univariate and multivariate logistic regression. They identified patient age, EGFR mutation status, distant metastasis and carcinoembryonic antigen level as significant risk factors for LM. Their model achieved an AUC of 0.767 in the test set (19). Hua et al. developed an NSCLC-LM risk prediction model incorporating five clinicopathological parameters: genetic mutation profile, primary tumour surgery, neurological symptoms, N stage and treatment strategy, with an internal-test AUC up to 0.861 (20). Yin et al. constructed a model integrating univariate and multivariate Cox regression to estimate LM risk and post-diagnosis survival in patients with LUAD. They revealed that EGFR/ALK mutation status, KPS score and extracranial metastasis were significantly associated with the prognosis of LUAD-LM, with the proposed model yielding a C-index of 0.64 in the validation cohort (21). Additionally, using Cox regression, Hong et al. developed a prognostic model for LM in patients with LUAD. The study identified KPS, distant lymph node metastasis, simultaneous diagnosis of lung cancer and LM and a lower neutrophil-to-lymphocyte ratio as independent prognostic factors, with a C-index of 0.71 (22). Collectively, the studies show that existing models provide a degree of predictive capability by integrating selected clinicopathological features; however, their reported AUC and C-index values indicate a substantial room for improvement. Moreover, existing models rely on a relatively narrow set of parameters and fail to apply a broader range of routinely collected clinical data and biochemical markers available in real-world settings. This explains the limitations to their predictive performance and applicability in clinical practice.

The Prompt model is superior to existing models because of the broad and diverse variables it incorporates. Beyond standard clinical and demographic characteristics, such as age, sex, smoking history and TTLS, the model integrates serum biochemical markers (e.g. ALB, GLB, Ca^2+^ and CO_2_), genomic mutation profiles, history of antitumour treatment and relevant clinical symptoms. This multidimensional variable set enables the Prompt model to capture a wider span of biological and clinical features that influence LM development in LUAD, thereby enhancing predictive performance and clinical utility. Notably, serum biochemical markers, as non-invasive and routinely obtained clinical measures, improve usability by decreasing patient burden and enhancing real-world deployment and clinical applicability (23). The dynamics of these biochemical markers allow clinicians to monitor disease fluctuations in real time and provide more responsive and accurate decision support, strengthening the utility of the Prompt model for early risk prediction and survival prognostication.

Our study identified several factors associated with increased LM risk in LUAD. Higher total CO_2_ on ABG analysis, longer TTLS, EGFR exon 21 L858R mutation and increased serum Ca^2+^ concentration were found to be risk contributors. In contrast, lower IBIL and higher KPS score were protective factors. As a key metabolic by-product of tumour activity, CO_2_ may influence the risk of LM by altering the tumour microenvironment through shifts in pH, hypoxia, acidosis and nutrient deprivation. In turn, tumour cells adapt to these stressors through metabolic reprogramming that fosters invasiveness and metastatic potential, thereby increasing the risk of LM (24, 25). Ca^2+^, a central intracellular second messenger, plays a role by activating CaMK and PKC signalling pathways that drive cytoskeletal remodelling and extracellular matrix degradation. Under micro-environmental stress, specific calcium channels facilitate Ca^2+^ influx, which, together with increased CO_2_, modulates an invasive cellular phenotype (26–28). These findings underscore the biological importance of Ca^2+^ and CO_2_ in LM progression and provide new mechanistic insights and potential molecular targets, exhibiting translational potential for early-stage risk detection and accurate treatment of LM.

In the present study, TTLS is defined as the time from the initial diagnosis of LUAD to the first clinical suspicion of LM. Our findings show a positive association between TTLS duration and LM incidence, indicating that patients with longer intervals from diagnosis to clinical suspicion demonstrate a higher subsequent risk of confirmed LM. Potential mechanisms responsible for this relationship are described as follows. First, prolonged TTLS may reveal a favourable response of the primary tumour to systemic therapy, leading to extended overall survival and longer temporal window, during which tumour cells may traverse the blood–brain barrier and seed the leptomeninges. Second, while modern treatments such as targeted therapy can achieve substantial control of systemic disease, many agents have limited penetration across the blood–brain barrier. This therapeutic pressure may render the CNS a relative ‘sanctuary site’, potentially facilitating LM development. Third, longer survival provides more time for clonal evolution, increasing the probability of emergent subclones with increased invasive or metastatic potential (29). These considerations suggest that patients with longer TTLS may benefit from more vigilant CNS monitoring despite otherwise well-controlled systemic disease. Future studies should validate these hypotheses, clarify the molecular mechanisms involved and explore potential preventive strategies.

Notably, previous studies have demonstrated that the EGFR exon 21 L858R mutation is a crucial predictor of brain metastasis in lung cancer, with higher mutation levels being correlated with an increased risk of metastatic spread to the brain (30). Consistent with these findings, our study identified a significant association between this mutation and the risk of LM. This may be attributable to the enhanced invasiveness and metastatic potential of tumour cells harbouring the mutation, such as a higher proportion of cells in the G2 phase, which reflects weakened cell-cycle arrest mechanisms (31).

In this study, the KPS score, which is a critical parameter for evaluating patients’ functional status, demonstrated substantial value in risk assessment and prognostication. KPS score was identified as a protective factor against a high LM risk among patients with LUAD. Higher KPS scores were associated with a lower probability of developing LM. Moreover, in the prognostic model, the KPS score was determined as an independent prognostic biomarker closely related to patient survival. Higher KPS scores indicate a more favourable prognosis. This may be explained by the fact that these patients can better tolerate treatment, maintain a higher quality of life and exhibit greater treatment adherence, ultimately improving OS. Therefore, the KPS score facilitates the identification of high-risk patients and provides clinicians with critical prognostic information, supporting the development of more precise treatment strategies and management plans.

Additionally, this study identified that decreased serum Na^+^ (<138.8 mmol/L) and Cl^-^ (<101 mmol/L) levels were significantly associated with poor prognosis in patients with LUAD-LM.

Beyond the mechanistic considerations discussed above, it is essential to critically interpret the prognostic biochemical markers identified in our Cox model. Serum Na^+^ and Cl^-^, as well as indirect bilirubin and Ca^2+^, may represent overlapping constructs rather than pure causal drivers of LM progression. First, these markers could be correlates of disease severity: more extensive CNS involvement may disrupt hypothalamic–pituitary regulation and impair osmotic homeostasis, leading to electrolyte derangements that simply reflect, rather than drive, poor outcomes. Second, they may capture treatment-related effects: targeted agents or chemotherapies can affect renal tubular function or ion channel activity, inducing secondary electrolyte shifts that confound the association with survival. Third, they may serve as proxies for broader systemic deterioration: hypoalbuminaemia, liver dysfunction (reflected by bilirubin), or metabolic acidosis (reflected by CO_2_) often accompany advanced cancer cachexia and multi-organ insufficiency. Thus, while our models demonstrate robust statistical associations, these biomarkers should be viewed as useful prognostic indicators and risk-stratification aids rather than direct therapeutic targets. Their clinical value lies in routine, non-invasive monitoring that may trigger earlier neurologic evaluation, rather than in establishing a causal chain amenable to specific biochemical manipulation.

The underlying mechanisms may involve multifactorial interactions. Tumour invasion into the hypothalamic or pituitary regions may induce dysregulated antidiuretic hormone secretion and impair osmotic regulation. Disruption of the blood–brain barrier can cause neuroendocrine imbalance, exacerbating electrolyte disturbances. Treatment-related factors, such as chemotherapy-induced nephrotoxicity and the effects of targeted agents on ion channels, may contribute to hyponatraemia and hypochloremia (32, 33). This association holds crucial clinical implications. Electrolyte abnormalities reflect the extent of CNS involvement and may also worsen cerebral oedema by altering CSF osmolarity, thereby aggravating neurological dysfunction (34). Dynamic changes in sodium and chloride levels are potential biomarkers for predicting therapeutic response. Incorporating routine electrolyte monitoring into the standardised assessment of patients with LM, combined with targeted interventions based on neurological symptoms, may provide prognostic value. Further studies are required to elucidate the molecular links between electrolyte homeostasis and the tumour microenvironment, with the aim of optimising therapeutic strategies.

Several limitations of this study should be acknowledged. First, the sample size is relatively modest, with only 129 real patients and 25 cases in the independent test cohort, which restricts the precision of point estimates for performance metrics. Given the limited test set size, we have prioritised calibration and decision curve analyses over reporting potentially unstable 95% confidence intervals for individual performance metrics. While the model shows consistent and favourable results across internal cross-validation, calibration assessment, and decision curve analysis, these findings remain exploratory in nature. Second, all participating centres are located in China. Variations in ethnic backgrounds, LM screening pathways and international therapeutic standards mean the generalisability of this leptomeningeal metastasis risk model to non-Chinese populations and foreign healthcare systems remains to be prospectively verified. Third, certain imaging and pathological parameters were not available for inclusion, and future work incorporating radiological features may further improve predictive performance. Furthermore, resampling algorithms including SMOTE will partially shift the inherent distribution of raw clinical data. Restricted by the low incidence of leptomeningeal metastasis and the limited number of authentic minority samples within the current cohort, comparative sensitivity analyses using alternative balancing methods were unfeasible. Additionally, the time point of clinical suspicion was determined retrospectively through medical record review, which may introduce minor recall bias. Prospective studies with predefined suspicion protocols will be valuable to validate the model’s real-time predictive performance. Therefore, based on real-world data from China, we developed and validated the Prompt model using the latest interpretable TabPFN machine-learning algorithm to evaluate risk factors for LM in patients with LUAD and predict their high-risk probability and survival outcomes. The model enables rapid identification of high-risk individuals using routinely available clinical variables. It emphasises total CO_2_ content on ABG analysis, TTLS, the EGFR exon 21 L858R mutation, serum Ca^2+^, IBIL and KPS score as key risk factors. To enhance interpretability and clinical applicability, we attempted to explain the model’s predictions and overcome the ‘black box’ limitations of machine learning using SHAP analysis. Furthermore, a web-based calculator was developed using the Python Streamlit framework to facilitate clinical implementation of the prediction model. The present study provides a valuable tool for early warning of high LM risk and for predicting survival in patients with LUAD-LM. The proposed model may help improve public awareness on health management, promote healthier lifestyles and support clinicians in more efficiently allocating limited medical resources. As an exploratory risk-stratification tool, the model may assist clinicians in prioritising patients for further neurologic workup, potentially reducing unnecessary diagnostic procedures in the future.

## Data Availability

The raw data supporting the conclusions of this article will be made available by the authors, without undue reservation.
