# Supplementary material for: Interpretable machine learning for prediction of leptomeningeal metastasis in lung adenocarcinoma: a multi-centre retrospective study via “Prompt” model
Source: Front Oncol. 2026 Sep 3;16:1894987. doi: 10.3389/fonc.2026.1894987 (PMC13581907; doi:10.3389/fonc.2026.1894987)
Supplement: Supplementary file 6 [file DataSheet6.pdf]

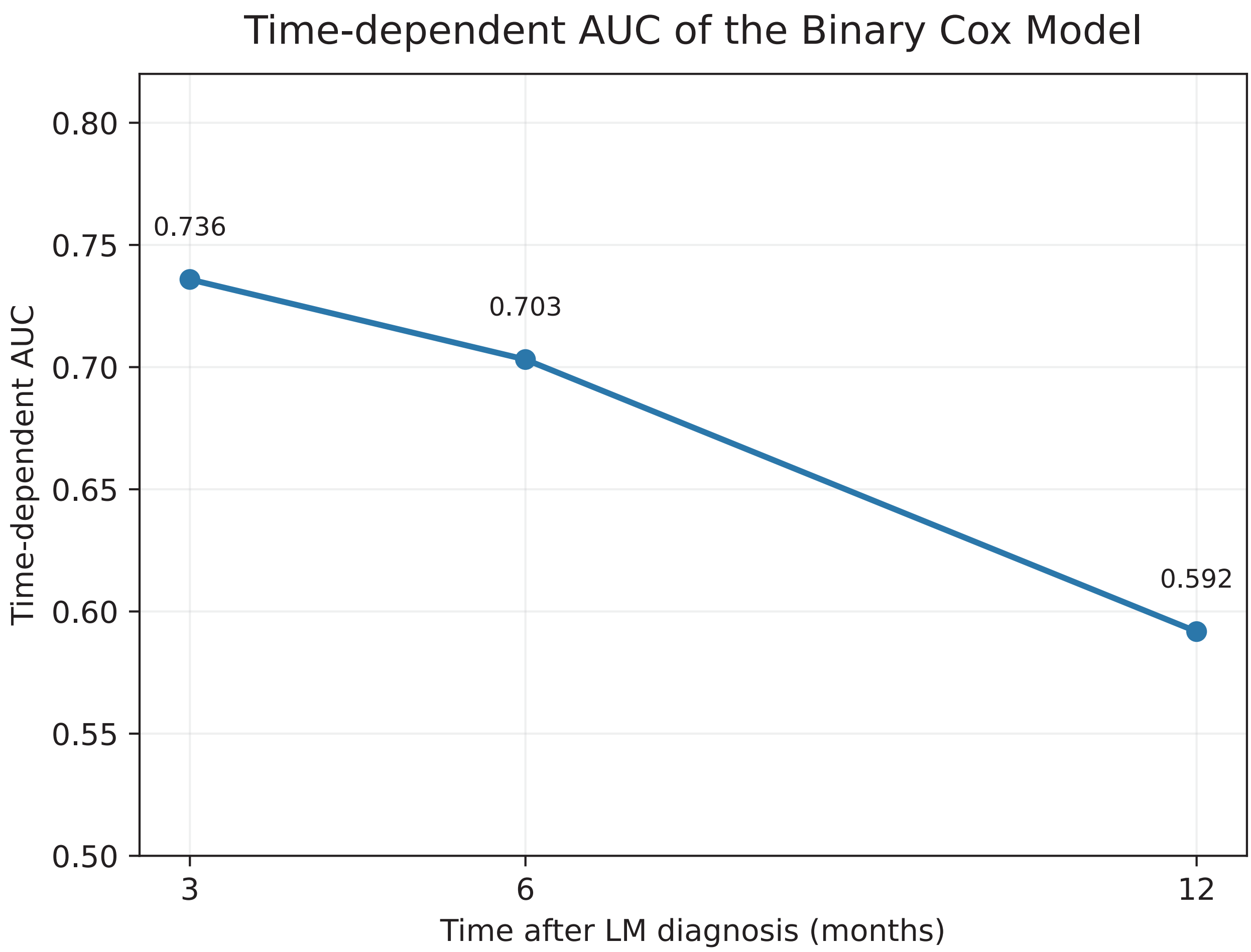

Figure S6. Time-dependent AUC of the Cox prognostic model at 3, 6, and 12 months.
